# Robot-assisted complete excision of choledochal cyst type I, hepaticojejunostomy and extracorporeal Roux-en-y anastomosis: a case report and review literature

**DOI:** 10.1186/1477-7819-8-87

**Published:** 2010-10-12

**Authors:** Thawatchai Akaraviputh, Atthaphorn Trakarnsanga, Nutnicha Suksamanapun

**Affiliations:** 1 Minimally Invasive Surgery Center, Division of General Surgery, Department of Surgery, Faculty of Medicine Siriraj Hospital, Mahidol University, Bangkok, Thailand; 2 Division of Pediatric Surgery, Department of Surgery, Faculty of Medicine Siriraj Hospital, Mahidol University, Bangkok, Thailand

## Abstract

For Choledochal cyst type I, complete excision of cyst with Roux-en-Y hepaticojejunostomy anastomosis is the treatment of choice. It has been performed laparoscopically with the advancement of laparoscopic skill. Recently, a telemanipulative robotic surgical system was introduced, providing laparoscopic instruments with wrist-arm technology and 3-dimensional visualization of the operative field. We present a case of robot-assisted total excision of a choledochal cyst type I and biliary reconstruction in a 14-year-old girl. No intraoperative complications or technical problems were encountered. An intraabdominal collection occurred and was successfully treated with continuous percutaneous drainage. At one-year follow-up, she is doing well without evidence of recurrent cholangitis.

## Background

Choledochal cyst is a rare congenital anomaly of the biliary system in the western countries, but has a higher rate of occurrence in Asia. This disorder is usually diagnosed during childhood and is more common in females. After being described first by Vater in 1723 [1], choledochal cysts are now classified using the Todani modification of the Alonzo-Lej classification system [2]. The most common is type I consisting of cystic, fusiform dilatation of the extrahepatic common bile duct. Untreated choledochal cysts are associated with complications such as recurrent cholangitis, acute pancreatitis and cholangiocarcinoma. The standard procedure is complete resection of the cyst with a Roux-en-Y hepaticojejunostomy anastomosis. Cystoenterostomy is no longer recommended [3]. Recently, many centers reported their experience with laparoscopic resection of the cyst [4]. Although this approach has been shown to be feasible and safe, most reports emphasized the technical challenge of the procedure as well as the long operative times [5]. The use of da Vinci Robotic Surgical System (Intuitive Surgical, Sunnyvale, California) provides the advantages of three-dimensional visualization through a stereoendoscope, tremor reduction, motion scaling, and wristed instrumentation with additional degrees of freedom compared to standard laparoscopic instruments [6,7]. We report the application of da Vinci Robotic Surgical System in type I choledochal cyst excision in a 14-year-old girl.

## Case presentation

**Figure 1:**
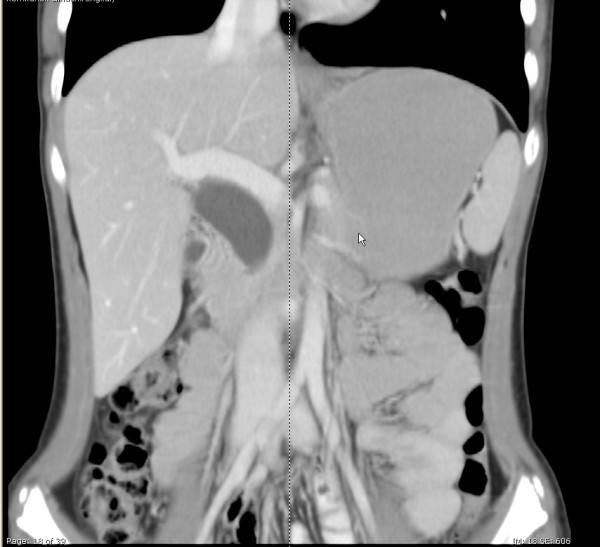
**Computed tomography scan demonstrating the choledochal cyst type I**.

A 14-year-old, girl presented with recurrent abdominal dyspepsia and intermittent jaundice. Her blood laboratory examinations were within normal limits. Serum CA 19-9 was normal. Ultrasonography demonstrated a large cystic dilatation of common bile duct. An abdominal computed tomography (CT) scan revealed a type I choledochal cyst measuring > 4 cm in diameter (Figure 1). The patient underwent da Vinci robot-assisted excision of the choledochal cyst, hepaticojejunostomy, and extracorporeal jejuno-jejunostomy of Roux-en-Y limb.

### Surgical technique

**Figure 2:**
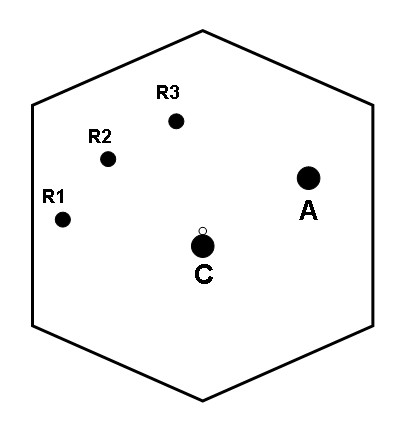
**Schematic illustration of the port placement: C, 12-mm camera port; R1-3, 8-mm robotic instrument ports; A, 12-mm assisted port**.

**Figure 3:**
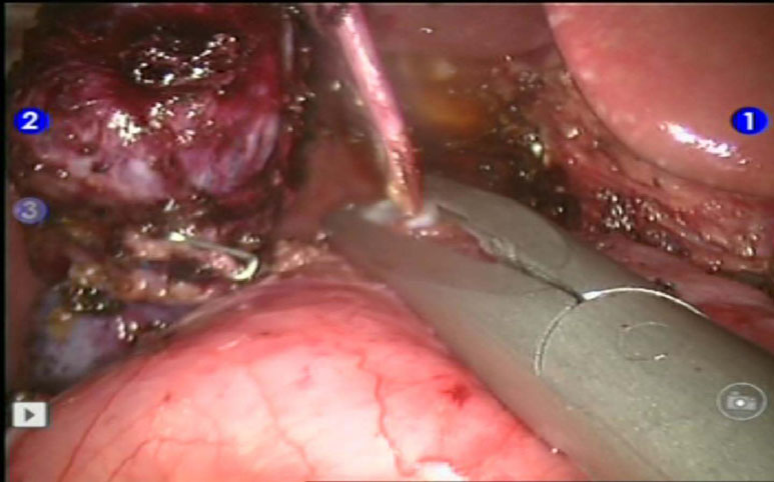
**Intraoperative finding of the narrow pancreatic part of common bile duct ligated with a plastic clip**.

The patient was placed in supine position. The pneumoperitoneum was created upto 12 mmHg using closed technique with Veress needle. Three 8-mm robotic trocars and two 12-mm trocars for camera and accessory device were applied (Figure 2). After introduction of the camera and wrist arm instruments, the table was placed in reverse Trendelenburg position to allow the intestines to fall caudaully. With the 3^rd ^robotic arm instrument, the liver was retracted more cephalad to better expose the porta hepatis. The portal dissection was begun firstly. The cyst was carefully dissected, preserving the hepatic arteries as well as the portal vein lying posterior to it. It was started on the inferior half of the cyst. Once the portal vein and hepatic arteries were separated from the cyst, the dissection was carried inferiorly toward the pancreas. The cyst was eventually found to taper rapidly to a small duct. The common bile duct was then ligated with plastic clips and transected (Figure 3). The cyst was then dissected cephalad until normal caliber common hepatic duct (CHD) was identified.

**Figure 4:**
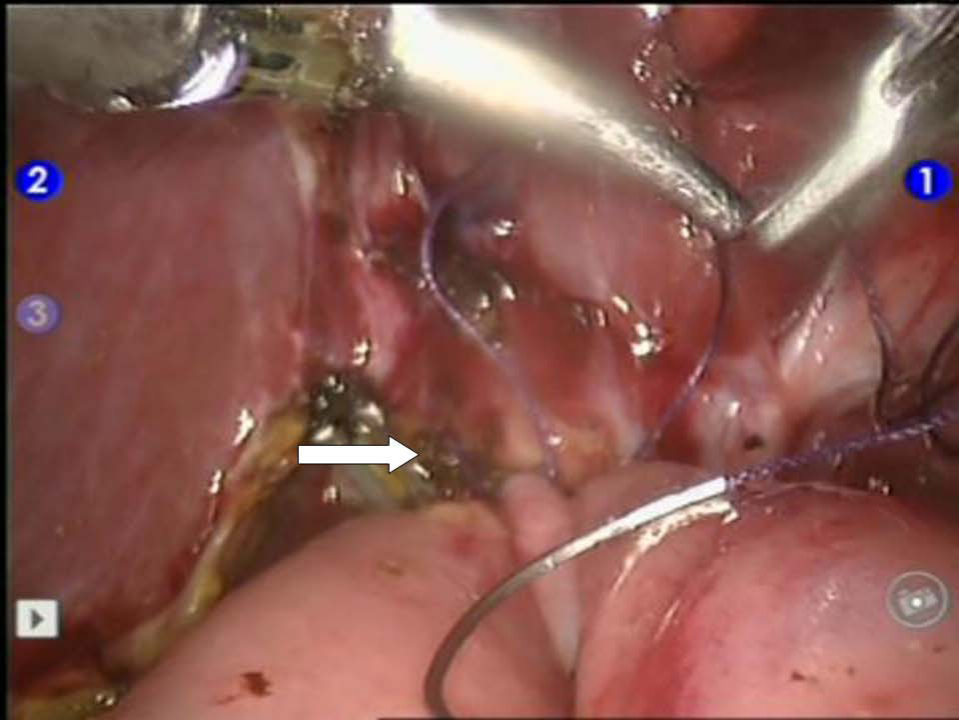
**The Robot-assisted end-to-side hepaticojejunostomy (white arrow) was completely performed with Vicryl #3/0 interrupted stitches**.

The gallbladder was dissected in top-down fashion. The cystic artery was clipped and divided. The CHD was transected and then complete cyst excision was done. The resected specimen was placed in right subdiapharmatic space. The jejunum was transected at about 20 cm from duodenojejunal junction by endo GIA staple. An end-to-side hepaticojejunostomy, anticolic route, was created using interrupted 3-0 Vicryl suture (Figure 4). After completion of the anastomosis, the robotic system was undocked and small upper midline incision was made. Side-to-side enteroenterostomy anastomosis was created outside abdominal cavity. The Roux-en-Y limb and jejunojejunostomy were re-checked and confirmed to be in good position without any evidence of torsion, bleeding, or bile leak. Jackson Pratt drain was placed. Finally the resected specimen was removed through this incision. The fascial and skin incisions were closed with absorbable sutures.

The total procedure time was 180 minutes. The total robotic setup time (preparation, port placement, docking) was 30 minutes and the total robotic operative time was 120 minutes. No intraoperative complications or technical problems were encountered.

### Postoperative course

One week after the operation, the Jackson Pratt drain was removed. Unfortunately she developed high fever and abdominal distension. CT scan revealed small right subdiaphramatic intraabdominal collection. Percutaneous drainage was performed with ultrasound guide and pigtail 7Fr silicone tube was placed. About 120 ml of clear yellowish color fluid was aspirated and bile leakage was diagnosed. Systemic antibiotic was applied. One week later, she had no fever and tolerated regular diet well. Pathological result confirmed choledochal cyst without evidence of malignancy. On postoperative 4th week, the tube was removed and she was discharged from the hospital. At one-year follow-up, she is doing well without any evidence of recurrent cholangitis.

### Discussion

Laparoscopic surgery has revolutionized the approach to abdominal surgery. Technological advancements have resulted in the application of minimally invasive techniques to increasingly complex procedures. However, standard laparoscopic approach of hepatobiliary surgery is still limited due to the technical complexities of these procedures. The rigid nature of the instruments with limited degrees of freedom, coupled with the fulcrum effect of laparoscopy and 2-dimensional imaging, certainly contributes to the limitations of the laparoscopic approach. Robotic technology may help overcome these obstacles.

The robot eliminates surgeon tremor and allows 3-dimensional visualization of the operative environment [2], which can allow the correct identification of anatomical variation. However, the main advantage of the da Vinci surgical system is the dexterity afforded by the Endowrist design, which allows precise control of technically challenging tasks such as delicate dissection, fine suturing [4]. It may be that advanced robotics will be reserved for only the most complex operations, such as choledochojejunostomy or pancreaticoduodenectomy. Robotic surgery can ameliorate the technical difficulties encountered laparoscopically and may allow surgeons to perform delicate procedures with shorter operative time [8-10].

Although robotic-assisted results and outcomes abound for many procedures, only limited information has been published on robotic-assisted choledochal cyst excision. We found only 4 cases in the literatures (Table 1). Interestingly, the Roux limb could be created entirely intracorporeally by the robot or extracorporeally through a small incision, which could decrease the robotic time and total operative times. In our case, we did an extracorporeal jejuno-jejunostomy anastomosis, and therefore our operative time was significantly shorter than the others report in literature. The minor leakage of hepatico-jejunostomy anastomosis found may be caused by unsecured suturing technique from the early experiences in robotic surgery.

**Table 1:** The summary of robotic-assisted choledochal cyst excision.

No	Author	Year	Age	Gender	Total OPT (min.)	No of port	Robotic time (min.)	Roux limb	LOH (day)	Complication
1	Woo et al.^11^	2006	5	F	440	5	390	Extracorporeal	4	no

2	CM Kang et al.^12^	2007	63	F	380	5	270	Extracorporeal	15	no

3	JJ Meehan et al.^7^	2007	2	N/A	445	5	408	Intracorporeal	N/A	no

4	JJ Meehan et al.^7^	2007	9	N/A	472	5	428	Intracorporeal	N/A	no

5	The study	2010	14	F	180	5	120	Extracorporeal	20	Collection

Disadvantages include the size of the robotic hardware in relation to patient body; the loss of haptic feedback; and the overall cost of the hardware, drapes, and maintenance of the robotic system. The robotic approach in gastrointestinal tract surgery has also a learning curve period regard to suturing technique, but we believe that this might be shorter than the standard laparoscopic surgery [11,12].

Finally, the robotic approach to the complex hepatobiliary surgery is feasible and safe in selected patients. Three-dimensional visualization, articulating instruments, and fine-motion filtering are the principle advantages. Robotic surgery may increase the variety of procedures, which can be accomplished with a minimally invasive approach and may also enable more general surgeons to perform these complex procedures. Surgeons need to become familiar with these improvements as the technology continues to progress [13].

## Conclusions

In summary, we report the feasibility and safety of robot-assisted laparoscopic resection of a type I choledochal cyst in a child. Compared to total laparoscopic surgery, the robot-assisted technique facilitates the most difficult part of the procedure, namely the creation of the hepaticojejunostomy anastomosis. Further experience is needed to properly evaluate the advantages and applicability of this approach, especially in the pediatric patient.

## Consent

Written informed consent was obtained from the patient for publication of this case report and any accompanying images. A copy of the written consent is available for review by the Editor-in-Chief of this journal.

## Competing interests

The authors declare that they have no competing interests.

## Authors' contributions

TA was the surgeon who performed the operation. TA and AT draft the manuscript. AT and NS participated in the operation. All authors read and approved the final manuscript.
